# Sirtuin 3 promotes microglia migration by upregulating CX3CR1

**DOI:** 10.1080/19336918.2019.1629224

**Published:** 2019-06-17

**Authors:** Runjing Cao, Shiping Li, Junxiang Yin, Li Guo, Jiong Shi

**Affiliations:** a Department of Neurology, The Second Hospital of Hebei Medical University, Shijiazhuang, China; b Barrow Neurological Institute, St. Joseph Hospital and Medical Center, Dignity Health Organization, Phoenix, AZ, USA; c Advanced Innovation Center for Human Brain Protection, Capital Medical University, Beijing, China; d China National Clinical Research Center for Neurological Diseases, Department of Neurology, Beijing Tiantan Hospital, Capital Medical University, Beijing, China

**Keywords:** Ischemia, SIRT3, microglia migration, CX3CR1

## Abstract

We studied the role of Sirtuin 3 (SIRT3) in microglial cell migration in ischemic stroke. We used a middle cerebral artery occlusion (MCAO) model of focal ischemia. We then applied lentivirus-packaged SIRT3 overexpression and knock down in microglial N9 cells to investigate the underlying mechanism driving microglial cell migration. More microglial cells appeared in the ischemic lesion side after MCAO. The levels of SIRT3 were increased in macrophages, the main source of microglia, after ischemia. CX3CR1 levels were increased with SIRT3 overexpression. SIRT3 promoted microglial N9 cells migration by upregulating CX3CR1 in both normal and glucose deprived culture media. These effects were G protein-dependent. Our study for the first time shows that SIRT3 promotes microglia migration by upregulating CX3CR1.

Stroke is the second most common cause of death and the third most common cause of disability worldwide [1]. After ischemic stroke, oxygen and energy depletion trigger inflammatory responses that leads to a cascade of cellular events such as glutamate excitotoxicity, oxidative stress and apoptosis [2]. Microglia constantly monitor the microenvironment in the central nervous system (CNS) [3]. Since this process must be kept efficient, microglia can be rapidly activated by pathological changes following ischemic stroke [4]. The migration of microglia is tightly regulated by cytokines. Fractalkine receptor (CX3CR1) is specifically expressed in microglia and macrophages. It is a Gi-protein coupled receptor and binds solely to CX3C ligand 1(CX3CL1, fractalkine) [5]. After ischemic stroke occur, CX3CR1-expressing microglia accumulate to the inflammatory sites [6].

Sirtuin 3 (SIRT3) plays a central role in maintaining mitochondrial function by limiting oxidative stress and reducing reactive oxygen species (ROS) production [7]. Its expression decreases with metabolic disease and neurodegenerative disease [8,9]. We have demonstrated that SIRT3 protects the brain from ischemic stroke by modulating superoxide dismutase 2 (SOD2) and the forkhead box O3a (FoxO3a) [10]. Since microglia are mainly involved in inflammatory responses of ischemia, this study investigates the role of SIRT3 played in the migration of microglial cells.

## Microglial cells were increased after ischemic stroke

**Figure 1.**
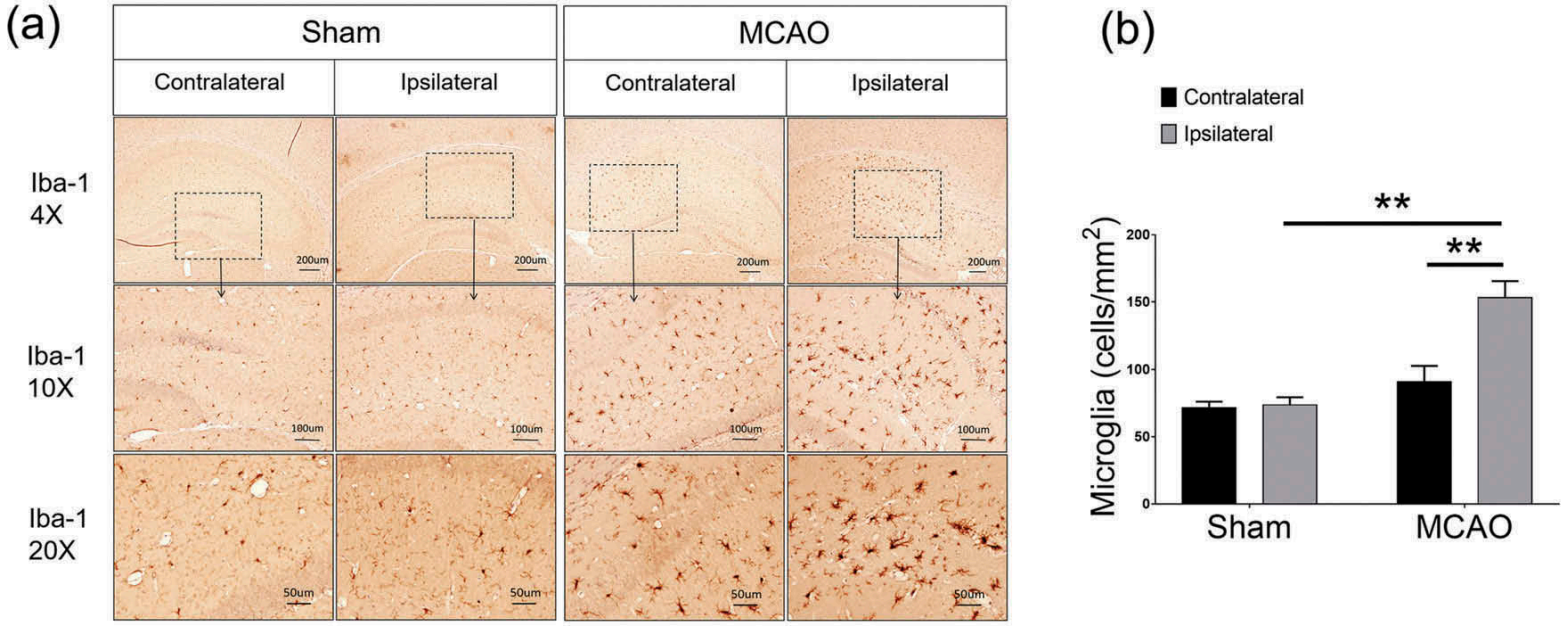
Microglial cells were increased after ischemic stroke. (a) Representative images showed that microglia increased in the ipsilateral side after MCAO. (b) Semi-quantification analysis of the number of Iba-1 positive cells. Data were presented as mean± S.E.M., *p < 0.05, **p < 0.01. Five random fields were counted for each sample, n = 5 per group.

Microglial cells in the brain were increased after MCAO. Compared with the contralateral side and sham-operated controls, microglia increased remarkably in the ipsilateral side (Ipsilateral 159.1 ± 11.03, vs. contralateral 90.97 ± 11.78 p < 0.01, vs. sham 71.62 ± 4.511 p < 0.01, Figure 1(a,b)). Moreover, these increased microglial cells in the ipsilateral side were in an active form, showing rods, amoeboid-like irregular shapes, while those in the sham-operated brains, were mainly in inactivated ramified forms (Figure 1(a)).

## SIRT3 levels were increased in macrophages after ischemic stroke

**Figure 2.**
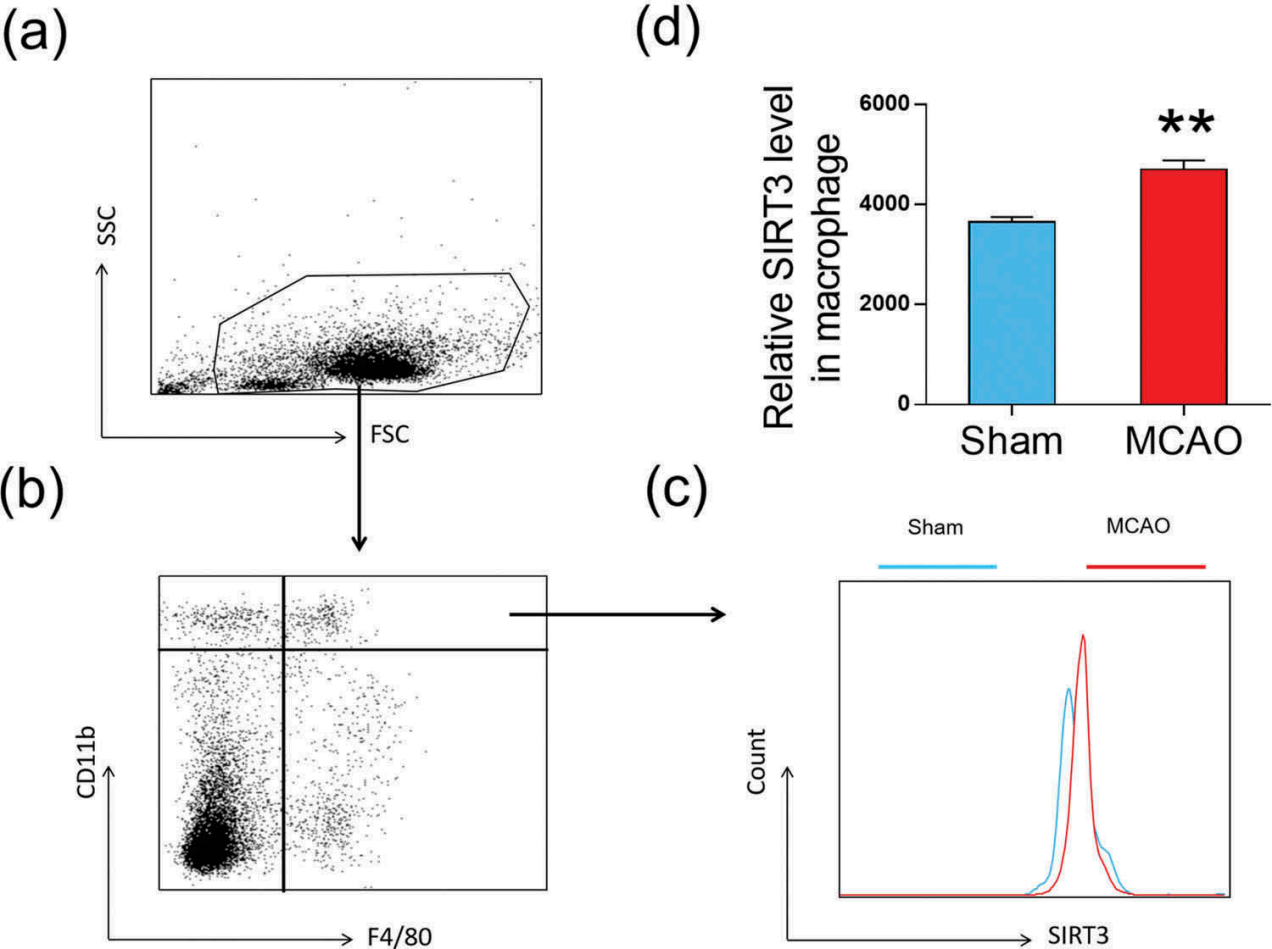
SIRT3 levels were increased in macrophages after MCAO (a) and (b) CD11b+ F4/80+ are used for gating macrophage cells. (c) Flow cytometry analysis is performed to measure SIRT3 levels in macrophage cells. (d) Bar graph demonstrates relative SIRT3 levels in macrophage cells were increased after MCAO. Data were presented as mean± S.E.M., **p < 0.01 compared with Sham group, n = 5 per group.

Since microglial cells are derived from peripheral macrophages, the increased microglia after MCAO are likely from the spleen where macrophages are produced. We measured SIRT3 levels in macrophages from the spleen. CD11b and F4/80 makers were used for gating macrophages (CD11b+F4/80+, Figure 2(a,b)). SIRT3 levels were measured in these macrophages and showed an increase after MCAO vs. sham (4703 ± 178.7 vs. 3652 ± 99, p < 0.01, Figure 2(c,d)).

## SIRT3 promoted microglial cell migration

**Figure 3.**
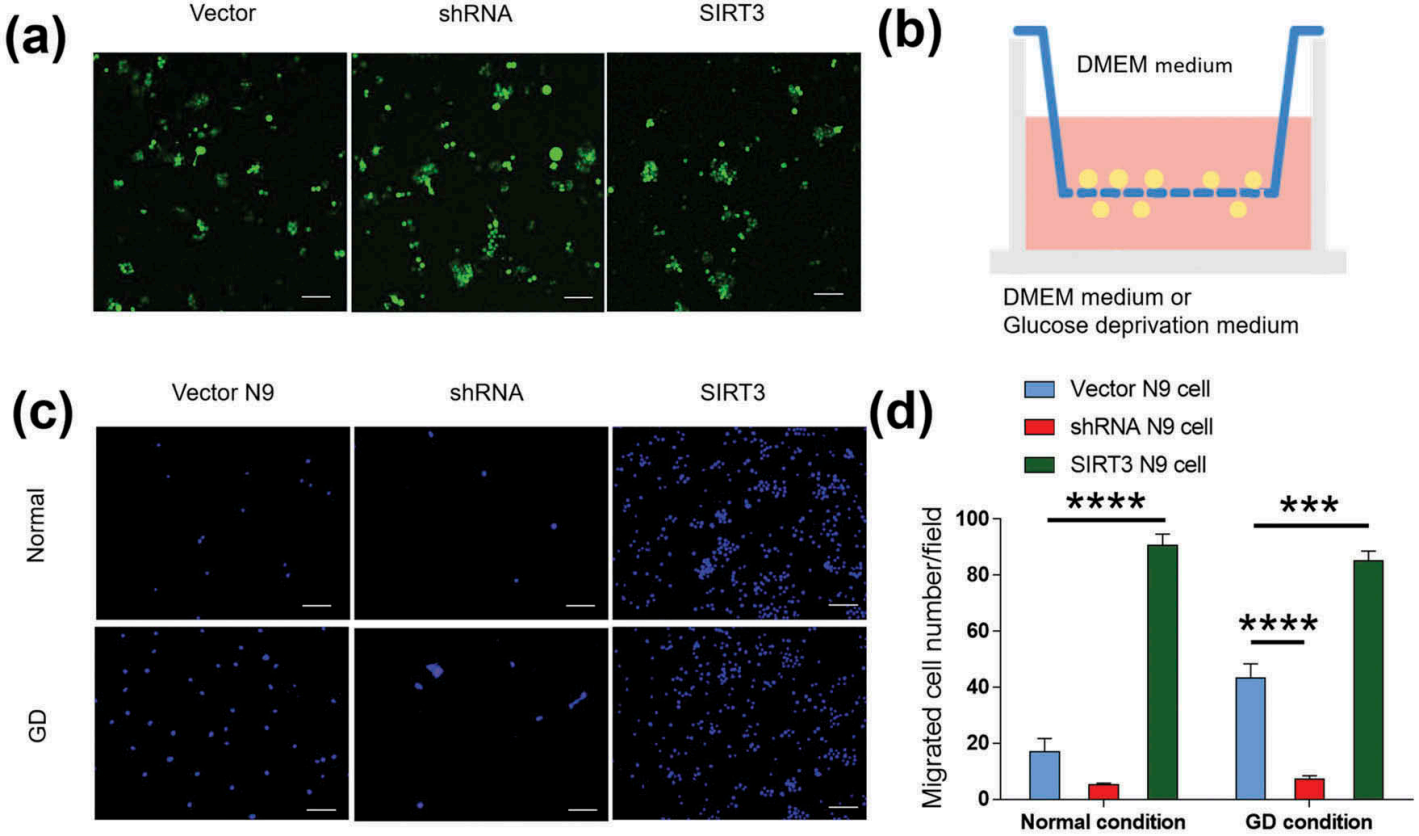
SIRT3 promoted the migratory ability of microglia. (a) Representative images illustrated that SIRT3 was overexpressed or knocked down in N9 cells. All Lenti-Virus vector containing the sequence of eGFP were visualized. Scale bars = 100 μm. (b)Schematic graph showed the plate structure for the migration assay. (c) Representative images showed that the migration of N9 cells transfected with Vector, shRNA and SIRT3 in normal or GD media. The migration of SIRT3 N9 cells increased compared with Vector N9 cells, and the migration of shRNA N9 cells decreased compared with Vector N9 cells both under normal or GD condition. Scale bars = 100 μm. Five random fields were counted for each group. Each experimental group was repeated three times. (d) Bar graph showed per field migrated cell numbers of N9 cells transfected with Vector, shRNA and SIRT3 in normal or GD media. Data were presented as mean± S.E.M., *p < 0.05, **p < 0.01, ***p < 0.001, ****p < 0.0001.

We then investigated whether SIRT3 promoted migration of microglia to the ischemic brain by genetic manipulation. We overexpressed and knocked down SIRT3 in N9 cells (Figure 3(a)) and performed the migration assay (Figure 3(b)). Under normal conditions, SIRT3 stimulated the migratory ability of N9 cells. Overexpression of SIRT3 increased N9 migration (90.6 ± 4.106 SIRT3 N9 cell vs. 16.89 ± 4.855 Vector N9 cell, p < 0.0001, Figure 3(c,d)); whereas knocking down of SIRT3 decreased N9 cell migration (5.2 ± 0.5831 shRNA N9 cell, Figure 3(c, d).

Glucose deprivation (GD) is an in vitro model of ischemia [11]. We placed the GD medium on the bottom of the Transwell plate to stimulate N9 cells to migrate to the lower chamber. N9 cells increased their mobility under the GD condition compared with the normal condition (43.2 ± 5.152 GD condition vs. 16.89 ± 4.855 normal condition, p < 0.01, Figure 3(c,d). This is consistent with the result we have seen in the MCAO model. Under glucose deprivation condition, with SIRT3 genetic manipulation, overexpression of SIRT3 increased N9 migration (85.2 ± 3.426 SIRT3 N9 cell vs. 43.2 ± 5.152 Vector N9 cell, p < 0.001, Figure 3(c,d)). Not surprisingly, knocking down of SIRT3 had the opposite effect (7.167 ± 1.276 shRNA N9 cell vs. 43.2 ± 5.152 Vector N9 cell, p < 0.0001, Figure 3(c,d)).

## SIRT3 regulated microglial cell migration via G protein-dependent CX3CR1

**Figure 4.**
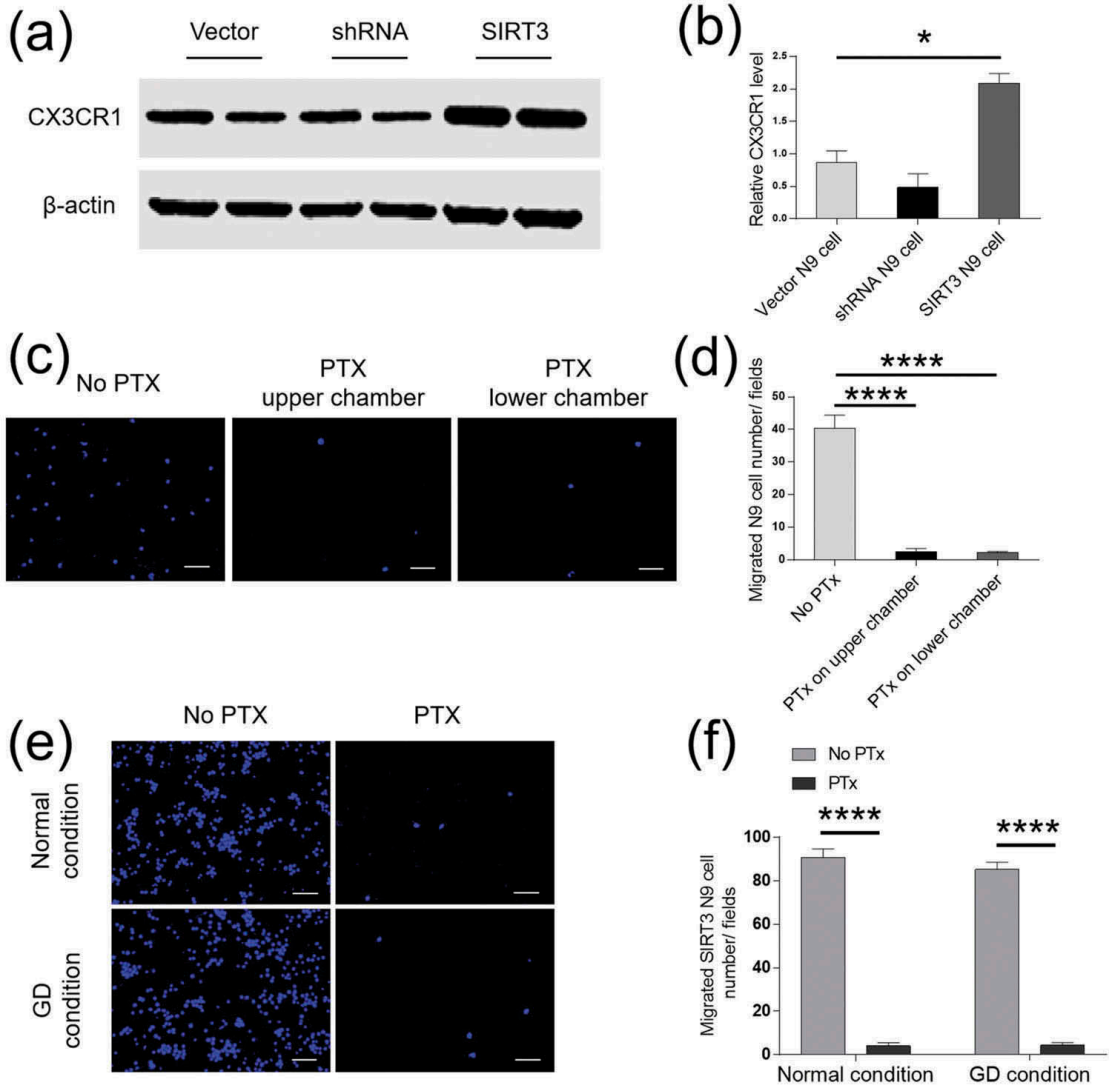
SIRT3 regulated microglial cell migration via G protein-dependent CX3CR1. (a) Representative western blots of CX3CR1 were shown in vectors or SIRT3 transfected cells. CX3CR1 levels increased in SIRT3 N9 cells compared with Vector N9 cells. n = 5 per group. (b) Bar graph showed that the density of CX3CR1 in N9 cells transfected by Vector, shRNA or SIRT3 Lenti-virus. β-actin was an internal control. (c) Representative images illustrated the migration of Vector N9 cells after adding PTx to the culture medium. PTx can block the migration of N9 cells by administrated on upper or lower chamber. Scale bars = 100 μm. Five random fields were counted for each group. Each experimental group was repeated three times. (d) Bar graph showed the migrated cell numbers per field with and without PTx. (e) Representative images showed that migration of SIRT3+ N9 cells was blocked by PTx in both the normal and GD media. Scale bars = 100 μm. Five random fields were counted for each group. Each experimental group was repeated three times. (f) Bar graph showed the migrated SIRT3+ N9 cell numbers per field after adding PTx in the normal and GD media. Data were presented as mean± S.E.M., *p < 0.05, **p < 0.01, ***p < 0.001, ****p < 0.0001.

CX3CR1 is expressed on microglial surface and controls the ability of microglial cell migration. To understand whether a change in SIRT3 expression affected CX3CR1 levels, we overexpressed or and knocked down SIRT3 in N9 cells. CX3CR1 levels were changed accordingly. Overexpression of SIRT3 increased CX3CR1 levels (2.087 ± 0.15 SIRT3 N9 cell vs. 0.8694 ± 0.1784 Vector N9 cell, p < 0.05) by more than two folds; whereas knockdown of SIRT3 reduced CX3CR1 levels by half (0.484 ± 0.2077 shRNA N9 cell vs. 0.8694 ± 0.1784 Vector N9 cell, Figure 4(a,b)). Since CX3CR1 is G protein-dependent, we added PTx, a G protein blocker in the medium of the upper or lower chambers of the Transwell plate. We found that PTx dramatically blocked the migration of N9 cells (2.6 ± 0.9274 PTx in the upper chamber, p < 0.0001; 2.167 ± 0.4773 PTx in the lower chamber, p < 0.0001 vs. 40.29 ± 4.156 no PTx, Figure 4(c,d)). There was no difference in N9 cell migration between PTx in the upper chamber and in the lower chamber. PTx was then added into the upper chamber for the following experiments. The normal DMEM medium or the GD medium was separately added into the lower chamber. We found that in the presence of PTx, the migration of N9 cells was reduced in both the DMEM medium and the GD medium (under normal condition: 90.6 ± 4.106 no PTx versus 4 ± 1.517 PTx, p < 0.0001; under GD condition: 85.2 ± 3.426 no PTx versus 4.4 ± 1.208 PTx, P < 0.0001, Figure 4(e,f)), suggesting a G protein-dependent effect.

In the present study, we have demonstrated for the first time that SIRT3 promotes microglial cell migration by increasing G protein-dependent CX3CR1 levels. Microglial cells are the main participant in inflammatory reaction, they play a key role in ischemic outcomes. After ischemia, microglial cells were activated and attracted to ischemic region. Previous studies showed peripheral inflammatory cells including blood-borne macrophages were recruited to the ischemic regions in the brain [12]. Further studies reported that activated microglial cells rapidly migrated into the infarction area at day 1 and reached maximal numbers at day 10 after transient focal cerebral ischemia [12–14]. Our study confirmed that increased microglial cells migrated to the ischemic region at 24 hours after MCAO.

SIRT3 has received much attention for its role in aging, neurodegenerative disease, cancer genetic, and stress resistance. However, whether SIRT3 participates in inflammatory reaction of ischemia is still unknown. We found that SIRT3 expression was increased in macrophages after ischemia, indicating SIRT3 takes part in modulating macrophage migration. Furthermore, SIRT3 promotes microglial N9 cell migration by upregulating CX3CR1.

CX3CR1, as a chemokine receptor, is named due to its ability to react with the attraction from CX3CL1 [15–17]. Once CX3CR1 is activated by CX3CL1, the intracellular calcium is elevated and triggers a cascade of secondary signaling that leads to microglial cytoskeletal rearrangement to promote migration [18]. Pertussis toxin (PTx) is a multisubunit protein toxin secreted by Bordetella pertussis, which inhibits G protein-coupled receptor signaling through G_i_ proteins in mammalian cells. It is widely used to block G_i_– dependent cellular events. Previous study showed that microglia migration could be diminished and blocked in the presence of PTx by targeting G_i_ proteins on CX3CR1 [18]. In this study, we have found that SIRT3 increases G protein-dependent CX3CR1, which brings more activated microglial cells to the ischemic region.

Microglial cells serve both detrimental and beneficial roles in ischemia at different stages of the disease course [19–21]. Some reported that excessively activated microglia damaged neurons by secreting neurotoxic factors such as ROS and inflammatory cytokines [20,21]. By contrast, other studies showed that selective elimination of microglia results in augment of neuronal death after MCAO in mice [22]. A future study that uses Cre/loxP mouse models which selectively deplete SIRT3 in microglia or neurons will allow us to investigate the intricate relationship between SIRT3 and microglia/neurons.

In summary, our study demonstrates that SIRT3 promotes microglial cell migration by upregulating CX3CR1. It provides us with new insights in developing new therapeutic targets for ischemic stroke.

## Animals

C57BL/6 male mice (age 10–14 weeks) were purchased from the Jackson Laboratory (Bar Harbor, ME, USA). All animals were housed in a temperature and humidity-controlled vivarium with free access to food and water and kept on a 12-hour dark/light cycle. Twenty mice were equally divided into two groups: middle cerebral artery occlusion (MCAO) group or a sham-operated control group. All experimental procedures were approved by the Institutional Animal Care and Use Committee of the Barrow Neurological Institute and performed according to the Revised Guide for the Care and Use of Laboratory Animals.

## Middle cerebral artery occlusion model

The mouse was anesthetized and prepared in a sterile manner. Its neck area was incised to expose the right internal carotid artery. A 6–0 nylon monofilament was inserted into the right internal carotid artery to block the bifurcation of the right internal carotid artery and MCA to achieve occlusion. After 60-minute of occlusion, the monofilament was removed to allow reperfusion. During the surgery, standard aseptic surgical procedures were applied. Moreover, the animals were kept warm with a carefully monitored heating pad to maintain body temperature at 37°C throughout the surgery and recovery after surgery. After surgery, mice were housed one per cage for recovery. Food and water were available on the floor of the cage. Mice were monitored daily for any sign of infection and distress. Antibiotics and analgesics can be applied if needed.

Neurological score was assessed after MCAO induced stroke [23]: 0 = no deficit; 1 = forelimb weakness; 2 = circling to affected side; 3 = partial paralysis on affected side; 4 = no spontaneous motor activity. Mice who has a neurologic score more than 2 points were chosen for the MCAO group.

## Immunohistochemistry

Mice were euthanized with isoflurane anesthesia at 24 hours after MCAO. They were perfused intracardiacally with saline followed by 4% paraformaldehyde for 30 minutes. After the brain was fixed, we embedded the brain in paraffin and cut it into serial 6 µm thick coronal slides. The antibody used for labeling microglia is anti-Iba-1 (019–19741, Wako, Japan). Immunolabeling was detected by applying the peroxidase-antiperoxidase procedure with 3,3ʹ-diaminobenzidine as a co-substrate. A microscope (Bx53; Olympus America Inc., Center Valley, PA, USA) was used to collect digital images under the bright field setting. Five random fields were counted for each sample, and each group includes five samples.

## Spleen cell isolation and flow cytometry

Single-cell suspensions (10^6^ cells) were isolated from spleen tissue to isolate macrophage [24]. Mice were anesthetized and perfused to remove peripheral blood at 24 hours after MCAO and sham operative-surgery. Each group contained five mice. The spleen was isolated and ground in PBS by mechanical trituation through a 70 -μm cell strainer (Corning, New York, NY, USA). The cell suspension was then centrifuged for 5 minutes at 1,000 rpm at 4°C. The supernatant was discarded. The cells were resuspended in red blood cell lysis buffer (Biolegend, San Diego, CA, USA) for 5 minutes to remove red blood cells. The cell suspension was centrifuged again for 5 minutes at 1,000 rpm at 4°C, and the supernatant was discarded. After being washed in PBS, the remaining cells were resuspended in 100 ul of PBS and stained with fluorochrome-conjugated antibodies. The following antibodies were used: PE-cyanine7 conjugated CD11b antibody (25–0112-82, eBioscience, Waltham, MA, USA), APC-cy7 conjugated F4/80 (123,117, Biolegend, San Diego, CA, USA). SIRT3 (sc-99,143, Santa Cruz Biotechnology, Dallas, TX, USA). Alexa Fluor® 488 Goat Anti-Rabbit IgG (H + L) (A11034, Waltham, MA, USA). Flow cytometry data were acquired on a FACSAria flow cytometer (BD Biosciences, San Jose, CA, USA) and analyzed using Flowjo 7.6 software (Informer Technologies, Ashland, OR, USA).

## Sirtuin 3 vector construction and transfection

A microglia cell line (N9) (American Type Culture Collection, Manassas, VA, USA) was cultured for in vitro study. DMEM medium or Earle’s without glucose medium were used to culture these cells. The medium was partially replaced every 4 days. We overexpressed and knocked down SIRT3 on N9 cells. Exogenous mouse SIRT3 cDNA sequence was subcloned into Lenti-CMV-GFP vector to overexpress SIRT3. A short sequence of 19 nucleotides targeting SIRT3 location 764 into OmicsLink small hairpin RNA (shRNA) expression clone was constructed to knock down the expression of SIRT3 [9]. We packaged the shRNA vector, Lenti-SIRT3 vector and a control vector into third-generation Lenti-Virus transfection system. Because eGFP was contained in all vectors, the effectiveness of the transfection can be visualized after adding the virus particles for 4 days. The levels of knockdown and overexpression of SIRT3 was also quantified by Western blotting as previously reported [9].

## Western blotting

For the *in vivo* studies, brain tissues of the ischemic core, penumbra and contralateral side were collected after the MCAO and sham surgery. Brain tissues were weighted and homogenized in a fresh cold buffer to extract the total tissue proteins. Fifty micrograms of the total tissue protein were used for western blotting. For the *in vitro* studies, cells were homogenized in a fresh cold cell lysis buffer (Cell Signaling, Danvers, MA, USA) and the total protein was extracted. Each experimental group contained five samples. Fifty micrograms of the total protein were loaded for Western blotting. Immunoreactive signals were quantified using Odyssey Fc (LI-COR Biosciences, Lincoln, NE, USA). Protein levels were presented relative to β-actin. The following antibodies were used: anti- SIRT3 (Cell Signaling, Danvers, MA, USA), anti-β-actin (Santa Cruz, Dallas, TX, USA), anti-CX3CR1 (Thermo Fisher Scientific, Waltham, MA, USA), IRDye 800CW and IRDye 680RD antibodies (LI-COR Biosciences, Lincoln, NE, USA).

## Transwell migration assay

A Transwell (pore size 8-mm, Corning, VWR, San Dimas, CA) assay was used to further analyze cell migration according to the manufacturer’s protocol. Microglia suspension (0.1 ml, 5 × 10^4^ cell/per well) was placed in upper chamber and incubated with or without PTx (100 ng/ml, Campbell, CA) in the DMEM/F-12 media. Earle’s without glucose or DMEM/F-12 media were added into the lower chamber. Cells were allowed to transmigrate into the lower chamber for 24 hours at 37°C. Cotton pads were used to clean the upper surface of the membrane. The membranes were then fixed with 4% paraformaldehyde in PBS for 30 minutes and stained with DAPI for 20 min. The migration of microglia was quantified and compared among the groups by counting the number of cells that migrated to the bottom side of the membrane. Five random fields at a 10X field were counted for each condition under a confocal microscope. Each experimental group was repeated three times.

## Statistical analysis

The results are presented as the means ± S.E.M. Statistical differences between two groups were evaluated by the two-tailed unpaired Student’s t-test. One-way analysis of variance (ANOVA) was used to determine the significance of multiple groups. Values of p < 0.05 were considered significant.
